# PPARα and PPARγ are expressed in midbrain dopamine neurons and modulate dopamine- and cannabinoid-mediated behavior in mice

**DOI:** 10.21203/rs.3.rs-2614714/v1

**Published:** 2023-03-02

**Authors:** Zheng-Xiong Xi, Briana Hempel, Madeline Crissman, Sruti Pari, Benjamin Klein, Guo-Hua Bi, Hannah Alton

**Affiliations:** National Institute on Drug Abuse; National Institute on Drug Abuse

## Abstract

Peroxisome proliferator-activated receptors (PPARs) are a family of nuclear receptors that regulate gene expression. Δ^9^-tetrahydrocannabinol (Δ^9^-THC) is a PPARg agonist and some endocannabinoids are natural activators of PPARa and PPARg. Therefore, both the receptors are putative cannabinoid receptors. However, little is known regarding their cellular distributions in the brain and functional roles in cannabinoid action. Here we first used RNAscope *in situ* hybridization and immunohistochemistry assays to examine the cellular distributions of PPARα and PPARγ expression in the mouse brain. We found that PPARα and PPARγ are highly expressed in ~70% midbrain dopamine (DA) neurons and in ~50% GABAergic and ~50% glutamatergic neurons in the amygdala. However, no PPARα/γ signal was detected in GABAergic neurons in the nucleus accumbens. We then used a series of behavioral assays to determine the functional roles of PPARα/γ in the CNS effects of Δ^9^-THC. We found that optogenetic stimulation of midbrain DA neurons was rewarding as assessed by optical intracranial self-stimulation (oICSS) in DAT-cre mice. Δ^9^-THC and a PPARγ (but not PPARα) agonist dose-dependently inhibited oICSS, suggesting that dopaminergic PPARγ modulates DA-dependent behavior. Surprisingly, pretreatment with PPARα or PPARγ antagonists dose-dependently attenuated the Δ^9^-THC-induced reduction in oICSS and anxiogenic effects. In addition, a PPARγ agonist increased, while PPARa or PPARγ antagonists decreased open-field locomotion. Pretreatment with PPARa or PPARγ antagonists potentiated Δ^9^-THC-induced hypoactivity and catalepsy but failed to alter Δ^9^-THC-induced analgesia, hypothermia and immobility. These findings provide the first anatomical and functional evidence supporting an important role of PPARa/g in DA-dependent behavior and cannabinoid action.

## Introduction

In 2020, over 14,000 American adults self-reported cannabis use disorder^1^. However, recreational legalization efforts continue to progress; in the last two years alone, 5 states have passed legislation allowing non-medical use of marijuana^2^. In this social and legislative climate, a full understanding of cannabis action and the underlying neural mechanisms is critically important. Δ^9^-tetrahydrocannabinol (Δ^9^-THC) is the primary phytocannabinoid within cannabis that is responsible for its subjective effects and many of its therapeutic benefits, which are widely believed to be mediated by activation of cannabinoid type 1 (CB1) and type 2 (CB2) receptors^3–6^. In addition to CB1 and CB2 receptors, Δ^9^-THC and other cannabinoids have high binding activity at other receptor sites such as the G protein-coupled receptor 55 (GPR55), the transient receptor potential cation channel (TRPV1), and the peroxisome proliferator-activated receptor gamma (PPARγ) and possibly alpha (PPARα)^5, 7, 8^. Evaluating the non-CB1 and non-CB2 receptor mechanisms underlying cannabinoid action will not only increase our understanding of cannabinoid biology but may also lead to the discovery of new interventions for treating cannabis dependence.

In this context, PPARs are of special interest due to their involvement in a number of CNS functions such as pain^9^, reward^10^, neuroinflammation^11^, and learning and memory^12^. Furthermore, the PPARγ agonist pioglitazone, an FDA-approved medication for the treatment of diabetes in humans, has been shown to be highly effective in reducing voluntary alcohol and opioid consumption and alcohol or nicotine-taking behavior in experimental animals^13–16^. However, the neural mechanisms underlying pioglitazone action are poorly understood.

PPARs are transcription factors within a subfamily of nuclear hormone receptors^17^. They are activated by lipophilic compounds and can bind directly to PPAR response elements, which are selective DNA sequences in target genes^11, 18^. The PPAR family contains three isoforms: PPARα, PPARγ, and PPARβ/δ – each with distinct physiological roles^19^. Recent work has identified interactions between these nuclear receptors and the endocannabinoid system. For instance, the synthetic cannabinoid WIN55,212–2 promotes transcriptional activity at both PPARα and PPARγ, as do the endocannabinoids 2-arachidonoyl-glycerol (2-AG) and anandamide^20–24^. As mentioned above, Δ^9^-THC binds to PPARγ, but findings regarding Δ^9^-THC’s affinity to PPARα are inconsistent. One report describes no binding affinity to PPARα^20^, while another reveals elevated transcriptional activity at PPARα in the presence of Δ^9^-THC^25^. No prior work has evaluated whether Δ^9^-THC binds to PPARβ/δ.

A small body of literature has emerged in the last two decades investigating the role of PPARs in cannabinoid activity outside of the CNS. For instance, in a neuronal cell culture model of Parkinson’s disease, Δ^9^-THC is neuroprotective and this response is blocked and reinstated by a PPARγ antagonist and agonist, respectively^26^. Additionally, both the tumor suppressant effect of Δ^9^-THC against liver cancer and its vasorelaxant response in the cardiovascular system are mediated by PPARγ activation^27,28^. However, no prior work has investigated whether PPARs underlie the CNS effects of cannabinoids and very little is known regarding the phenotypes of neurons that express PPARs in the brain.

To address these knowledge gaps, we first examined the cellular distributions of PPARα and PPARγ in multiple types of neurons in the midbrain ventral tegmental area (VTA), nucleus accumbens (NAc), and amygdala using double-staining RNAscope *in situ* hybridization (ISH) and immunohistochemistry (IHC) assays. Given their major distributions in midbrain dopamine (DA) neurons, we then used pharmacological approaches to manipulate PPARα and PPARγ and transgenic and optogenetic approaches to manipulate VTA DA neurons to determine the functional roles of PPARα and PPARγ in cannabinoid action and DA-dependent behavior.

## Materials And Methods

### Subjects

Male C57BL/6J mice (25–35 g; The Jackson Laboratory, Bar Harbor, ME) were utilized throughout the studies. Male and female DAT-Cre^+/−^ mice (25–40 g) were bred at the National Institute on Drug Abuse (NIDA) Intramural Research Program (IRP) and underwent genotyping by Transnetyx for verification. All subjects were kept on a reverse light cycle (lights off at 7:00 am; on at 7:00 pm) and provided with ad lib food and water. The house room temperature was set to 21–23°C with 40–50% humidity. Experimental procedures adhered to the *Guide for the Care and Use of Laboratory Animals, 8*^*th*^ *edition*. The Animal Care and Use Committee at NIDA approved the study protocol.

### Chemicals

Δ^9^-THC was provided by the NIDA pharmacy (Baltimore, MD). The stock solution was dissolved in ethanol at a concentration of 50 mg/ml. We diluted this solution as needed for experimental use in a 5% cremophor (Sigma-Aldrich, St. Louis, MO) saline solution. PPAR antagonists and agonists including GW9662, GW6471, pioglitazone, and GW7647 were purchased from Cayman Chemical (Ann Arbor, MI). Each compound was dissolved in a mixture of 2% DMSO, 3% tween-80 and 95% saline.

#### Experiment 1: RNAscope in situ hybridization

We first performed RNAscope *in situ* hybridization (ISH) to examine the distribution of PPARα and PPARγ mRNA in the mesolimbic DA system and amygdala – regions associated with the affective properties of cannabinoids. In the VTA, we examined PPARα (*PPARA*) and PPARγ (*PPARG*) mRNA expression in GABAergic (*GAD1*^+^), glutamatergic (*Slc17a6*^+^) and dopaminergic (*TH*^+^) neurons. In the NAc, we focused on PPAR expression in GABAergic (*GAD1*^+^) neurons, whereas in the amygdala, we looked at expression patterns in GABAergic (*GAD1*^+^) and glutamatergic (*Slc17a6*^+^) neurons. The complete RNAscope procedures are described in *Supplementary Information*.

#### Experiment 2: Immunofluorescence

RNAscope ISH assays detected weak PPARα and PPARγ mRNA signals. To better examine the expression of PPAR receptor proteins on different cell types in the VTA (GABA, glutamate, and TH), NAc (GABA), and amygdala (GABA & glutamate), we used double label immunostaining. The complete Immunofluorescence procedures are described in *Supplementary Information*.

#### Experiment 3: Optical Intracranial Self-Stimulation

In Experiment 2 we found that PPARα and PPARγ are highly expressed in midbrain DA neurons. To understand the functional role of these receptors we next examined how pharmacological manipulation of PPARα and PPARγ altered DA-dependent behavior in the presence or absence of Δ^9^-THC action in an oICSS paradigm described below. The complete oICSS procedures are described in *Supplementary Information*.

#### Experiment 4: Conditioned Place Preference or Aversion (CPP/CPA)

We then examined whether pretreatment with PPARα or PPARγ antagonists altered the aversive subjective effects of Δ^9^-THC using the CPP test. The complete CPP/CPA procedures are described in *Supplementary Information*.

#### Experiment 5: Elevated Plus Maze

Next, we considered the role of PPARα and PPARγ in Δ^9^-THC-induced anxiety in the elevated plus maze (EPM). The complete EPM procedures are described in Supplementary Information.

#### Experiment 6: Open field Locomotion

In this experiment, we first examined whether PPAR agonists or antagonists alter open-field locomotion by themselves, and then examined whether pretreatment with PPAR antagonists block Δ^9^-THC-induced hypoactivity. The complete locomotor test procedures are described in *Supplementary Information*.

#### Experiment 7: D^9^-THC-induced Tetrad

Lastly, we looked at whether PPARα and PPARγ mediate the classical tetrad effects produced by high doses (10, 30 mg/kg) of D^9^-THC. The complete tetrad experimental procedures are described in *Supplementary Information*.

### Statistical Analyses

All data are presented as means ±SEM. oICSS and tetrad data were analyzed based on changes in the area under the curve (ΔAUC) to better visualize group differences. Data were converted to ΔAUC by summating the difference between each time point after drug injection and a baseline value before the injection. One-way or two-way repeated measures (RM) ANOVAs were used to analyze the data as appropriate. Significant effects were followed by post hoc tests using Tukey’s multiple comparisons. For all tests, statistical significance was set to *p*<0.05.

## Results

### Cellular distributions of PPARα and PPARγ in the VTA, NAc, amygdala

**Figure 1:**
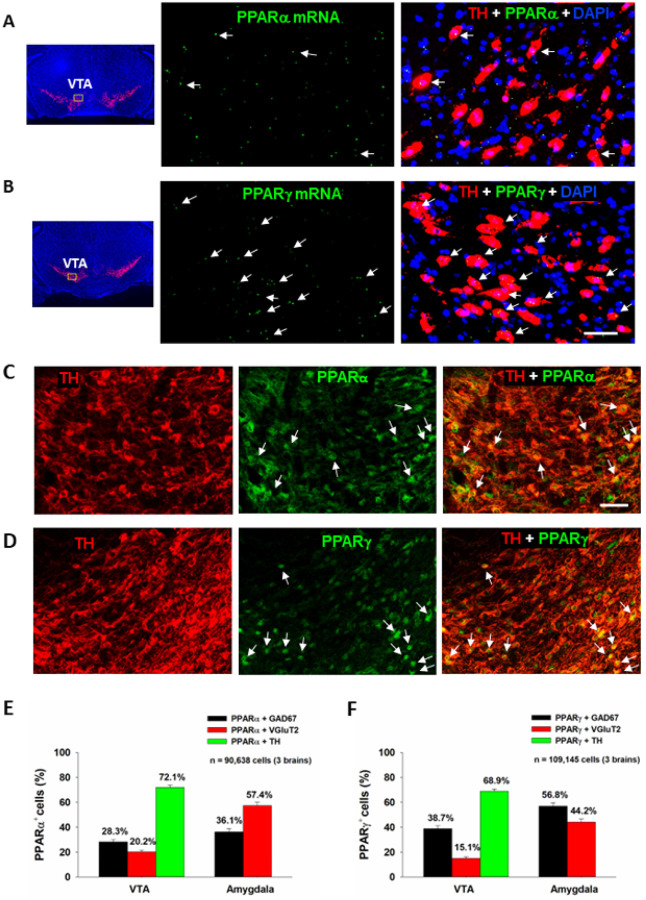
PPARα and PPARγ RNAscope ISH and immunostaining results. **A/B**: Representative RNAscope ISH, illustrating low densities of PPARα (**A**) and PPARγ (**B**) mRNA signals detected in TH^+^ DA neurons in the VTA. **C/D**: Representative images indicating that PPARα- or PPARγ-immunostaining was co-localized with TH-immunostaining in VTA DA neurons. **E/F**: The cell counting data indicate that PPARα and PPARγ are expressed in ~70% of DA neurons in the VTA and in 40~60% of GABA or glutamate neurons in the Amygdala. The scale bar indicates 50 mm. Each bar illustrates the average percentage of cells co-expressing PPARα or PPARγ with one neuronal marker (TH, GAD67 or VGluT2) out of the total number of DA, glutamate or GABA neurons. N = 3 brains with 5–6 slices selected from each brain and 2–4 images taken per region/slice. (see Figs. S1-S5 for PPARα or PPARγ mRNA or immunostaining in other types of neurons in the VTA, NAc and amygdala). The scale bar indicates 50 mm.

We first examined the expression of PPARα and PPARγ in different neuronal phenotypes in the mesolimbic DA system and amygdala, which are critical brain regions involved in cannabinoid action^5^. Figure 1 (A, B) highlights a representative image of PPAR mRNA staining, illustrating that PPARα and PPARγ mRNA are present in VTA DA neurons. Notably, more DA neurons displayed TH and PPARg colocalization than DA neurons showing TH and PPARa colocalization. PPARα and PPARγ mRNA was also detected in GABA and glutamate neurons in the VTA, NAc and amygdala (Fig. S1, S2). However, in these cell types, PPARα and PPARγ mRNA expression levels were low and observed outside of DAPI-labeled nuclei, complicating cell counting analyses. As such, cell counting was not attempted on these data.

The low PPARα and PPARγ mRNA expression levels observed in DA, GABA and glutamate neurons were unexpected given previous work demonstrating a strong neuronal signal using qPCR^29^. To address this discrepancy, we utilized a different technique, double-label IHC, to measure protein expression of PPARα and PPARγ in the predominant cell types within the regions of interest. We detected strong PPARα and PPARγ immunostaining in TH^+^ DA neurons in the VTA (Fig. 1 – C, D) as well as in GAD67^+^ GABA neurons and VgluT2^+^ glutamate neurons in the VTA and amygdala (Fig. S3, S4). In the NAc, no PPAR immunostaining overlapped with GAD67 + GABA neurons (Fig. S5). Surprisingly, PPARα and PPARγ immunostaining was detected mainly in astrocyte-like cells in the NAc, suggesting that these may be glial receptors. Quantitative cell counting assays revealed that PPARα and PPARγ are expressed in ~ 70% of DA neurons, ~ 30% of GABA neurons and ~ 20% of glutamate neurons in the VTA (Fig. 1 – E, F). In the amygdala, PPARα is found in ~ 60% of glutamate neurons and ~ 40% of GABA neurons, while PPARg is expressed in ~ 60% of GABA neurons and ~ 40% of glutamate neurons. In the NAc, PPARα/g and GAD67 co-expression was negligible, so no quantification was performed.

### PPARa/g modulate DA-dependent oICSS and Δ^9^-THC action in oICSS

We have recently reported that optogenetic stimulation of VTA DA neurons is rewarding as assessed by optical ICSS (oICSS) and real-time place preference^30, 31^ and this effect is dose-dependently attenuated by cannabinoids such as Δ^9^-THC, WIN55212,2 or AM-2201^32^. However, the receptor mechanisms underlying cannabinoid reward-attenuation in oICSS are unclear. Given that Δ^9^-THC is also a potent PPARg agonist (EC_50_ = 0.3 mM) and other cannabinoids have binding affinity to PPARa^33^, we first examined whether PPAR agonists produce similar effects as Δ^9^-THC and whether pretreatment with PPAR antagonists would block Δ^9^-THC-induced changes in oICSS in transgenic DAT-Cre mice.

**Figure 2:**
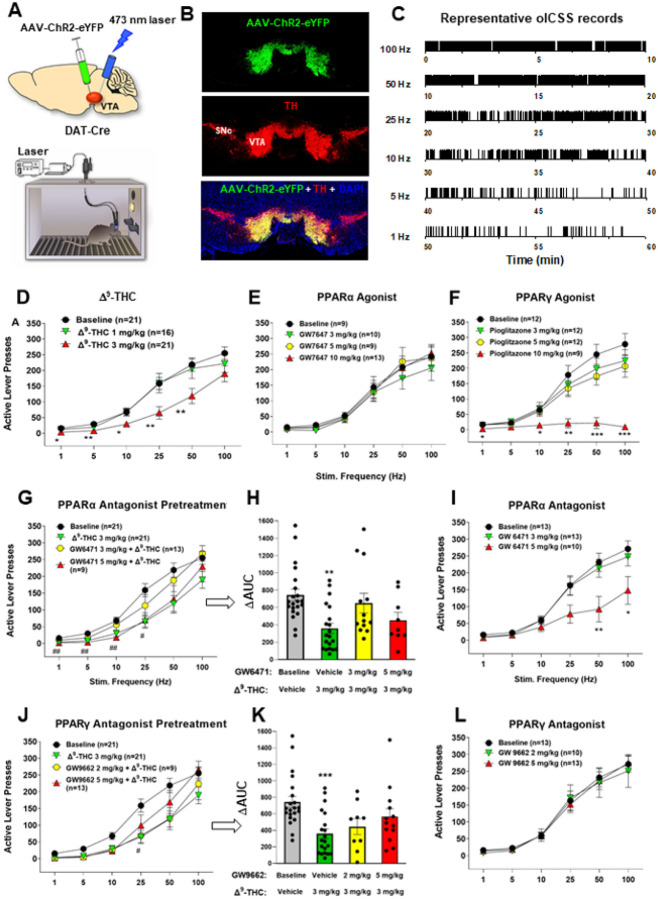
Effects of Δ^9^-THC and/or PPAR agonists and antagonists on optical brain-stimulation reward (oICSS) in DAT-Cre mice. **A:** Diagrams showing the general experimental methods. The AAV-ChR2-eYFP vectors were microinjected bilaterally into the midbrain VTA and two optical fibers were targeted to the VTA. Mice were placed in oICSS chambers and trained to press an active lever to obtain laser stimulation reward. **B:** Representative images showing AAV-ChR2-eYFP expression in TH^+^ DA neurons in the VTA. **C**) Representative lever responding to different frequencies of laser stimulation in a single session from a single mouse. **D:** Stimulation–response curve of lever responding over different frequencies of laser stimulation. Δ^9^-THC (1, 3 mg/kg, intraperitoneal, i.p.) dose-dependently shifted the oICSS curve downward compared with the vehicle (baseline) control group. **E/F:** PPARγ agonism (by pioglitazone) produced a similar inhibitory effect on oICSS as Δ^9^-THC, while PPARα agonism (by GW7674) failed to alter basal oICSS. **G/H:** Pretreatment with GW9662 (a selective PPARγ antagonist) dose-dependently attenuated Δ^9^-THC-induced reduction in oICSS. **I**) GW9662 alone failed to alter oICSS. **J/K:** Pretreatment with GW6471 (a selective PPARα antagonist) attenuated Δ^9^-THC-induced reduction in oICSS. L) GW6471 alone dose-dependently decreased oICSS responses. *p<0.05, **p<0.01, ***p<0.001 relative to baseline.

Figure 2 shows the experimental results, indicating that bilateral stimulation of VTA DA neurons maintains robust oICSS behavior in a stimulation frequency-dependent manner (Fig. 2 – A, B, C), which is dose-dependently inhibited by systemic administration of Δ^9^-THC (Fig. 2D) or pioglitazone (a PPARg agonist, EC_50_ = 0.69 μM, Fig. 2F), but not by GW7647 (a selective PPARa agonist, EC_50_ = 6 nM, Fig. 2E). A two-way RM ANOVA revealed a Significant Δ^9^-THC treatment main effect (Fig. 2D, F_2,49_ = 5.19, p < 0.01) and pioglitazone treatment main effect (Fig. 2F, F_3,41_ = 8.15, p < 0.001), but a non-Significant effect with GW7647 (Fig. 2E, F_3,37_ = 0.44, p > 0.05). More detailed statistical analysis results are shown in supplementary Table 1. This finding that a PPARγ, but not PPARα, agonist produces a Δ^9^-THC-like effect in oICSS suggests that Δ^9^-THC may inhibit brain-stimulation reward by activation of PPARγ.

To test this hypothesis, we then determined whether the PPARα antagonist GW6471 alters Δ^9^-THC-induced changes in oICSS. We found that pretreatment with GW6471 Significantly attenuated Δ^9^-THC-induced reduction in oICSS, with a lower dose of GW6471 being more effective in attenuation of Δ^9^-THC’s action (Fig. 2 – G, H). A two-way RM ANOVA revealed a Significant GW6471 treatment main effect (Fig. 2G, F_3,60_ = 3.79, *p* < 0.05). Unexpectedly, GW6471 itself produced a dose-dependent reduction in oICSS (Fig. 2I, F_2,33_ = 4.58, p < 0.05) whereas the PPARa agonist GW7647 failed to alter oICSS (Fig. 2E), suggesting that PPARα may be fully occupied and activated by endogenous ligands. Thus, the antagonist GW6471 may produce a reduction in oICSS by blockade of endogenous ligand binding to PPARa, while the agonist GW7647 may not work due to a ceiling effect caused by endogenous ligand binding.

**Figure 3:**
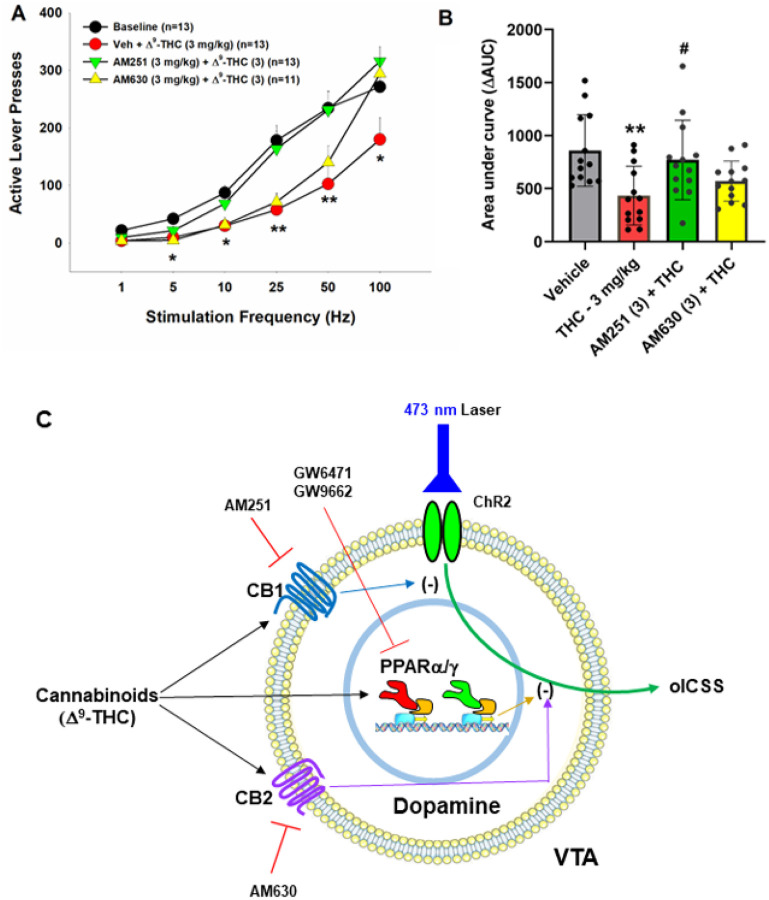
Effects of CB1 and CB2 receptor antagonists on Δ^9^-THC-induced changes in oICSS in DAT-cre mice. **A:** The stimulation-rate response curves showing that 3 mg/kg D^9^-THC Significantly decreased oICSS, which was blocked by AM251 and partially reduced by AM630. **B:** The DAUC data from the data in (A), illustrating that the reduction in oICSS by D^9^-THC was blocked by AM251 and partially reduced by AM630. C: A summary diagram showing how D^9^-THC modulates oICSS and how CB1, CB2 and PPAR antagonists block D^9^-THC action in oICSS. **p<0.01, ***p<0.001, relative to baseline. ^#^*p*<0.05, relative to D^9^-THC group.

Next, animals were pretreated with a PPARγ antagonist (GW9662). We found that GW9662 dose-dependently attenuated Δ^9^-THC-induced reduction in oICSS (Fig. 2 – J, K). Two-way RM ANOVAs over time (stimulation frequency) revealed a statistically Significant GW9662 treatment main effect (Fig. 2J, F_3,59_ = 5.80, *p* < 0.01). Analyzing the changes in the area under curve (DAUC) values for the data shown in Fig. 3G also revealed a Significant GW9662 pretreatment main effect (Fig. 2K, one-way ANOVA, F_2,54_ = 8.26, *p* < 0.001). Figure 2L shows that administration of GW9662 alone failed to alter oICSS (F_2,33_ = 0.04, *p* = 0.96). More detailed statistical analysis results are shown in supplementary Table 1. These findings provide the first behavioral evidence indicating that PPARa and PPARγ receptor mechanisms at least in part underlie Δ^9^-THC-induced reward attenuation.

We have previously reported that both CB1 and CB2 receptors are expressed in midbrain DA neurons and glutamate neurons^34–37^, which have been thought to play an important role in cannabinoid action^5, 38,39^. To provide a point of comparison for our PPAR findings, we examined the effects of AM251 (a selective CB1R antagonist) and AM630 (a selective CB2R antagonist) on Δ^9^-THC-induced changes in oICSS. Figure 3 shows that AM251 pretreatment almost completely blocked Δ^9^-THC suppression of oICSS (Fig. 3B, F_3,34_=5.76, p < 0.01), while AM630 partially reduced Δ^9^-THC activity. This data suggests that CB1R (and CB2R to a lesser extent) are involved in Δ^9^-THC’s aversive effects (Fig. 3C).

### Effects of PPAR antagonists on Δ^9^-THC-induced place aversions

Next, we examined whether pretreatment with PPAR antagonists is able to block Δ^9^-THC-induced conditioned place aversion (CPA) (Fig. S6-A). Figure S6 (B, C) shows that pretreatment with either the PPARa antagonist (GW6471) or PPARγ antagonist (GW9662) failed to alter Δ^9^-THC-induced CPA, suggesting that PPARs are not critically involved in Δ^9^-THC-induced place aversion. This is consistent with our previous reports that CB1 and CB2 receptor mechanisms underlie the rewarding and aversive effects^40, 41^. A two-way RM ANOVA on CPP scores in subjects administered Δ^9^-THC detected a Significant main effect of Test (cocaine CPP) (Figure S6-B, F_1,21_ = 13.74, p < 0.01), but not GW6471 dose (F_2,21_ = 0.06, *p* = 0.95) or the interaction between these factors (F_2,21_ = 0.007, *p* = 0.99). An identical analysis on CPP scores in subjects administered a PPARγ inhibitor showed a main effect of Test (Figure S6-C, F_1,21_ = 16.7, p < 0.001), but no GW9662 dose effect (F_2,21_ = 0.60, *p* = 0.56) or interaction (F_2,21_ = 0.09, *p* = 0.91).

We also examined the effects of the PPAR antagonists alone in CPP. We found that the PPARα antagonist GW6471 (Fig. S6-D, F_2,21_ = 1.21, *p* = 0.32) failed to produce either CPP or CPA, while the PPARγ antagonist GW9662, at a low dose (2 mg/kg), produced Significant place aversion in the absence of Δ^9^-THC (Figure S6-E, F_1,21_ = 8.95, *p* < 0.01), suggesting that PPARγ tonically modulates brain reward function under physiological conditions.

### Blockade of PPARs attenuates Δ^9^-THC-induced anxiety

**Figure 4:**
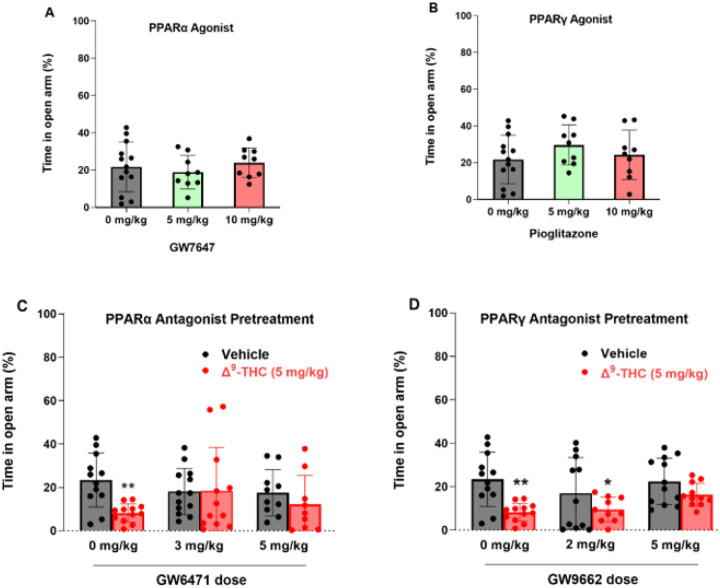
Effects of PPARα and PPARγ antagonists on Δ^9^-THC-induced anxiety in the elevated plus maze test. **A/B**: PPARα (GW7647) or PPARγ (pioglitazone) agonism produced neither anxiety nor anxiety relief. **C/D**: Pretreatment with PPARα (GW6471) or PPARγ (GW9662) antagonist attenuated Δ^9^-THC-induced anxiety. However, *p<0.05, **p<0.01, relative to vehicle. n = 9–13/group.

In addition to VTA DA neurons, PPARα and PPARγ are also expressed in 50 ~ 60% of GABA and glutamate neurons in the amygdala, a critical brain region involved in affective behavior. Therefore, we further examined the functional roles of PPARs in cannabinoid-induced anxiety. We first examined the effects of PPAR agonists in an elevated plus maze (EPM) test. We found that systemic administration of PPARα agonist (Fig. 4A, F_2,27_ = 0.67, *p* = 0.52) or PPARγ agonist alone (Fig. 4B, F_2,27_ = 0.73, *p* = 0.49) produced neither an anxiolytic nor anxiogenic response, as assessed by the times the animals spent on the open arm or closed arm of the EPM, respectively. However, pretreatment with either PPARa or PPARg antagonist Significantly attenuated Δ^9^-THC-induced anxiogenic effects (Fig. 4 – C, D), while PPARα or PPARγ antagonists alone failed to produce anxiogenic or anxiolytic effects (Fig. 4 – C, D, vehicle groups). These data suggest that PPAR mechanisms are critically involved in the anxiogenic effects of Δ^9^-THC. A two-way ANOVA on percent time in the open arm of the EPM showed a main effect of Δ^9^-THC dose (Fig. 4C, F_1,62_ = 4.706, *p* < 0.05), but not GW6471 dose (F_2,62_ = 0.41, *p* = 0.66) or the interaction between these factors (F_2,62_ = 2.26, *p* = 0.11). Post hoc comparisons revealed that Δ^9^-THC-induced anxiety is statistically Significant in the vehicle (0 mg/kg GW6471) control group. However, in subjects pretreated with 3 or 5 mg/kg GW6471 Δ^9^-THC did not produce Significant anxiogenic effects relative to vehicle control group (Fig. 4C). Another two-way ANOVA on Δ^9^-THC-induced anxiety produced a main effect of Δ^9^-THC dose (Fig. 4D, F_1,62_ = 18.93, *p* < 0.001), but not GW9662 dose (F_2,62_ = 1.25, *p* = 0.29) or the interaction term (F_2,62_ = 0.68, *p* = 0.51). Post hoc comparisons showed that subjects administered Δ^9^-THC by itself or in conjunction with 2 mg/kg GW9662 were more anxious relative to controls whereas the group given 5 mg/kg GW9662, and Δ^9^-THC did not produce Significant anxiogenic effects compared to the vehicle controls (Fig. 4D).

### Effects of Δ^9^-THC and PPAR antagonists on locomotor activity

**Figure 5:**
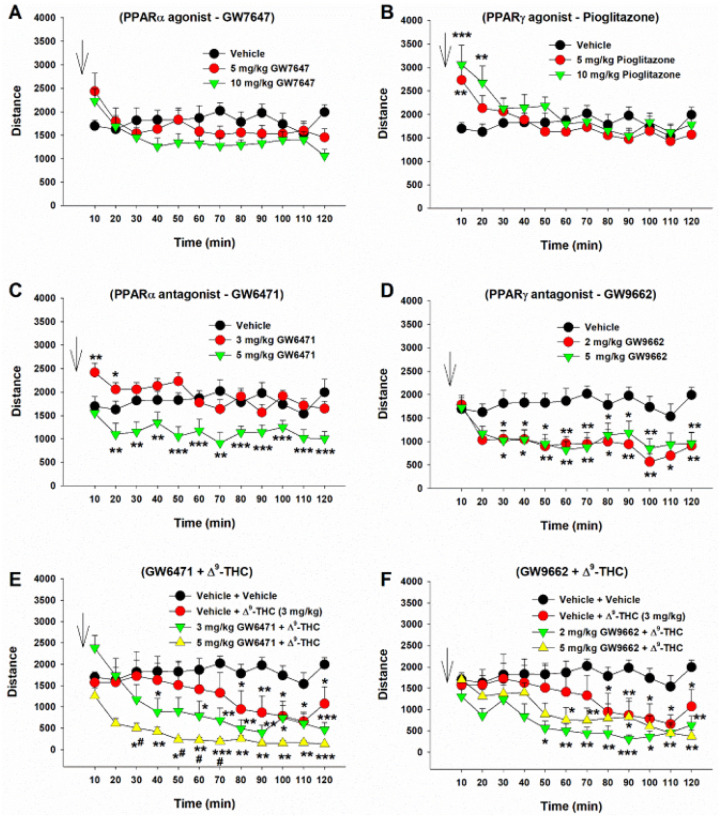
Effects of Δ^9^-THC and/or PPAR agonists or antagonists on open-field locomotion. **A/B:** Systemic administration of the PPARa agonist GW7647 failed to alter open-field locomotion (**A**), while the PPARg agonist pioglitazone produced a transient increase in locomotion (**B**). **C/D**: Systemic administration of PPARa antagonist GW6471 (**C**) or PPARg antagonist GW9662 (**D**) alone dose-dependently inhibited open-field locomotion. **E/F:** Pretreatment with GW6471 enhanced Δ^9^-THC-induced reduction in locomotor activity (**E**), while GW9662 pretreatment did not Significantly alter Δ^9^-THC action in locomotion (**F**). n = 8/group. * *p*<0.05, ** *p*<0.01, *** *p*<0.001, compared to the vehicle group. ^#^ *p*<0.05, compared to the (Vehicle + Δ^9^-THC) group (**E**).

We then examined the effects of Δ^9^-THC with or without ligands on open-field locomotion (Fig. 5). Systemic administration of a selective PPARa agonist (GW7647) failed to alter locomotor activity (Fig. 5A, F_2,21_ = 0.46, *p* > 0.05), while a selective PPARg agonist (pioglitazone) produced a Significant increase in locomotion, an effect that lasted for about 20 min. A two-way RM ANOVA did not reveal a Significant pioglitazone treatment main effect (Fig. 5B, F_2,21_ = 0.44, *p* = 0.65), but revealed a Significant treatment X time interaction (F22,231=5.36, *p* < 0.001). Post hoc group comparisons revealed a Significant increase in locomotion at 10 and 20 min after pioglitazone administration compared to the vehicle control group (Fig. 5B). In contrast, systemic administration of PPAR antagonists produced a Significant reduction in open-field locomotion (Fig. 5C, D). A two-way RM ANOVA reveal a Significant GW6471 treatment main effect (Fig. 5C, F_2,21_ = 17.39, *p* < 0.001) and a Significant GW9662 treatment main effect (Fig. 5D, F_2,14_ = 5.67, *p* < 0.01). More detailed statistical results are shown in the supplementary Table 2. These findings suggest that PPARg modulates basal locomotor behavior.

We then observed the effects of PPAR antagonist pretreatment on Δ^9^-THC-induced changes in locomotion. We found that systemic administration of 3 mg/kg Δ^9^-THC produced a Significant reduction in locomotion (Fig. 5 – E, F), consistent with our previous finding^42^. However, pretreatment with a selective PPARa antagonist (GW6471) enhanced Δ^9^-THC-induced hypoactivity (Fig. 5E), while a selective PPARg antagonist (GW9662) produced a trend toward an increase in Δ^9^-THC-induced reduction in locomotion. A two-way RM ANOVA revealed a Significant treatment X time interaction (Fig. 5E, F22,308 = 4.63, *p* < 0.001; Fig. 5F, F22,308 = 2.27, *p* < 0.001). The full statistical analysis results are shown in supplementary Table 2. These findings suggest that PPAR mechanisms may not underlie cannabinoid action in locomotion.

### Effects of PPARα/g antagonists on Δ^9^-THC-induced tetrad behavior

**Figure 6:**
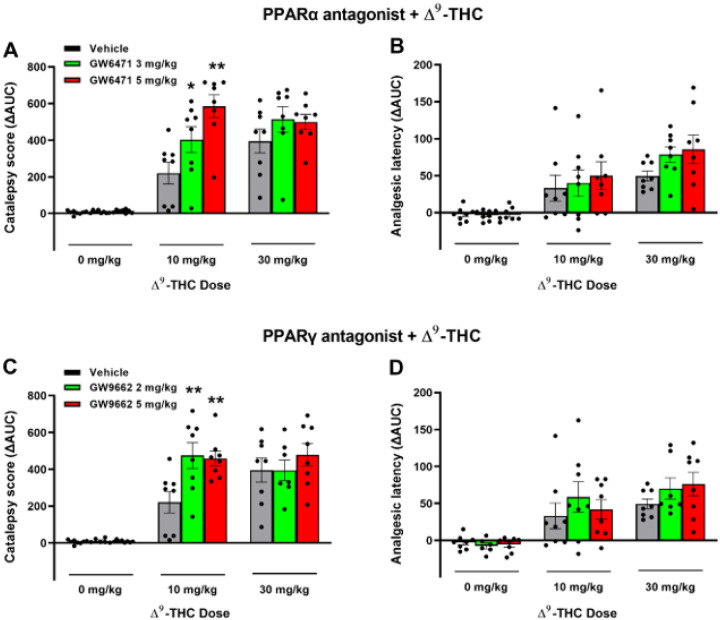
Effects of PPAR antagonists on Δ^9^-THC-induced catalepsy and analgesia in mice. **A/B:** Pretreatment with the PPARα antagonist GW6471 enhanced 10 mg/kg Δ^9^-THC-induced catalepsy (**A**), but did not Significantly altered hot-plate analgesia (**B**). **C/D:** Pretreatment with the PPARg antagonist GW9662 enhanced THC-induced catalepsy (**C**) but failed to alter Δ^9^-THC-induced analgesia (**D**). (See Fig. S7 and S8 for the effects of PPAR antagonists on Δ^9^-THC-induced hypothermia and immobility).

Lastly, we examined whether PPARs contribute to the classical tetrad effects of cannabinoids. Δ^9^-THC, at 10 and 30 mg/kg, produced prototypical cannabimimetic effects, e.g. catalepsy, analgesia, hypothermia, and rotarod locomotor impairment (i.e., immobility). The full time-course data are presented in Figures S7 and S8. To make the data easier to view and understand, we provide graphs utilizing the changes in area under curve (ΔAUC) values (Fig. 6). We found that pretreatment with the selective PPARa antagonist GW6471 produced dose-dependent enhancement in Δ^9^-THC-induced catalepsy (Fig. 6A), a trend toward an increase in Δ^9^-THC-induced analgesia (Fig. 6B), but no effect on Δ^9^-THC-induced hypothermia or immobility (Figs. S7, S8). A two-way RM ANOVA on the catalepsy ΔAUC data revealed a Significant main effect of Δ^9^-THC dose (Fig. 6A, F_2,21_ = 103.3, *p* < 0.001), GW6471 dose (F_2,21_ = 4.65, *p* < 0.05), and an interaction between these terms (F_4,42_ = 4.96, *p* < 0.05). Pairwise comparisons showed that Δ^9^-THC induced catalepsy at 10 mg/kg was Significantly enhanced by GW6471 (Fig. 6A). Similar two-way RM ANOVA’s were run for analgesia showing a Significant main effect of Δ^9^-THC dose (F_2,21_ = 23.06; *P* < 0.001), but not of GW6471 dose (F_2, 21_ = 1.51; *P* = 0.244) or the Δ^9^-THC × GW6471 interaction (F_4, 42_ = 0.55; *P* = 0.703). Additional two-way RM ANOVA results for the full-time course data (Fig. S7) are provided in the supplementary Table 3.

Similarly, pretreatment with a PPARγ antagonist (GW9662) enhanced the cataleptic effects of Δ^9^-THC but had no effect on Δ^9^-THC-induced analgesia, hypothermia and immobility (Fig. 6 – C, D; Fig. S8). A two- way RM ANOVA on catalepsy scores revealed a Significant Δ^9^-THC treatment main effect (Fig. 6C, F_2,21_ = 72.56, *p* < 0.001) and a Significant Δ^9^-THC X GW9662 interaction (F_4,42_ = 3.05, *p* < 0.05), although no GW9662 main effect (F_2,21_ = 3.15, *p* = 0.064). Post-hoc comparisons detected a Significant increase in 10 mg/kg Δ^9^-THC-induced catalepsy at both doses of GW9662 tested (2 & 5 mg/kg). Two-way RM ANOVAs on analgesic latency revealed Significant main effects of Δ^9^-THC dose (F_2,21_ = 20.54; *P* < 0.001), but not of GW9662 dose (F_2, 21_ = 0.78; *P* = 0.455) or GW9662 X Δ^9^-THC interaction (F_4, 42_ = 0.53; *P* = 0.716). Additional two-way RM ANOVA results for the full-time course data (Fig. S8) are provided in the supplementary Table 4.

## Discussion

The major findings in this report include: 1) PPARa and PPARg are mainly expressed on midbrain DA neurons, GABA and glutamate neurons in the amygdala, as well as on astrocyte-like cells in the NAc. 2) Optogenetic stimulation of VTA DA neurons is rewarding, which is dose-dependently inhibited by Δ^9^-THC and a PPARg, but not PPARa, antagonist, suggesting an important role of PPARg in DA-dependent behavior. 3) PPARa and PPARg antagonism attenuated the reward-attenuating (aversive) and anxiogenic effects of Δ^9^-THC and potentiated Δ^9^-THC-induced hypoactivity and cataleptic properties, but failed to alter Δ^9^-THC-induced analgesia, hypothermia and immobility. These findings implicate PPARa and PPARg in the VTA and amygdala in the affective profile of cannabinoids and DA-dependent behavior.

### PPARa and PPARg expression in dopamine, glutamate and GABA neurons

Prior studies using qPCR and IHC have localized PPARα to neurons, astrocytes, and microglia and PPARγ to neurons and astrocytes in both human and mouse brains and in cultured rat neurons^29, 43^. However, little is known about the phenotypes of neurons or cells that express PPARa and PPARg in the mesolimbic reward system and amygdala. In the present report, we detected PPARα and PPARγ immunostaining in ~ 70% of DA neurons in the VTA, with lower but detectable levels on VTA GABA and glutamate neurons, suggesting an important role of PPARs in modulating DA-dependent behavior. This is supported by our behavioral findings that activation of PPARg inhibited DA-dependent brain-stimulation reward as assessed by oICSS. Prior work has demonstrated that the PPARg agonist pioglitazone is effective in reducing feeding, voluntary alcohol consumption and drug self-administration^13–16^. As such, the present findings may implicate a dopaminergic PPARg mechanism in pioglitazone’s anti-reward effects.

Surprisingly, we detected PPARa and PPARg in accumbal astrocyte-like cells, but not on GABAergic medium-spiny neurons. This finding is inconsistent with previous reports in which PPARa/g-immunostaining was colocalized with primarily neuronal markers (NeuN or b-tubulin III), but not GFAP or Iba1 in the NAc and cortex^29, 43^. The reasons underlying these conflicting findings are unclear. However, it is important to note that in the present work we did not employ an astrocytic marker, but assumed based on anatomical similarities in our images. Further work is needed to address this question.

It was previously reported that PPARγ transcripts are detected in both the nucleus and cytoplasm of GABA neurons in the hippocampus and amygdala^44^. Cannabinoids have biphasic anxiolytic and anxiogenic effects, which are likely mediated by GABAergic and glutamatergic neurons in the amygdala, respectively^45, 46^. This inspired us to map out PPARa and PPARg expression in the amygdala and determine their preferred neuronal subtypes. PPARα was primarily expressed on glutamate neurons (57.3%) and PPARγ on GABA neurons (56.8%). These results are compatible with prior work and point to PPARs on both GABAergic and glutamatergic neurons in the amygdala as potential receptor mechanisms underlying the affective properties of cannabinoids.

We note that PPARa/g transcription levels by RNAcope ISH assays were fairly low in all three brain regions assessed and an unusual pattern of expression was observed such that individual puncta were distributed within and outside of DAPI-labeled nuclei. In previous reports, similarly low transcription levels and expression patterns have been noted in the amygdala and hippocampus^44, 47^. It is not clear why mRNA levels are deficient relative to PPARa/g-immunostaining. Further study is required to address this issue.

### PPARa/g activation contributes to Δ^9^-THC-induced aversion

We have previously reported that cannabinoids produce a reduction in NAc DA release and DA-dependent oICSS in transgenic DAT-cre or VgluT2-cre mice^32, 36, 41, 42^. However, the receptor mechanisms underlying cannabinoid action in oICSS have not been explored in the above studies. In the present study, we found that pretreatment with a CB1 (AM251) or CB2 (AM630) receptor antagonist Significantly blocked or reduced Δ^9^-THC-induced reduction in oICSS, suggesting that both membrane CB1 and CB2 receptors are critically involved in cannabinoid aversion. In addition to identification of CB1 and CB2 receptor expression in midbrain DA neurons^35, 36^, we also identified PPARa and PPARg in VTA DA neurons as discussed above. Furthermore, systemic administration of PPARg or pioglitazone (a selective PPARg agonist) inhibited oICSS, while pretreatment with a PPARγ antagonist Significantly weakened the suppressive effect of Δ^9^-THC in this assay. These findings suggest that PPARg activation may partially underlie Δ^9^-THC-induced reductions in oICSS. With PPARα, pharmacological activation failed to alter oICSS; however, pretreatment with a PPARa antagonist also reduced the suppressive effect of Δ^9^-THC, suggesting that PPARa may indirectly modulate Δ^9^-THC aversion via a non-dopaminergic mechanism. Together, these findings suggest that multiple receptor mechanisms, including membrane CB1 and CB2 and nuclear PPARs, underlie cannabinoid or Δ^9^-THC-induced reward-attenuation or aversion (Fig. 3C).

We note that blockade of PPARα/γ failed to alter Δ^9^-THC-induced place aversion. There are several possible explanations. First, Δ^9^-THC is not a selective PPARγ agonist. It also has binding activity at CB1, CB2 and GPR55 receptors^5, 7^. Thus, it is likely that Δ^9^-THC-induced place aversion is mediated by activation of multiple cannabinoid receptors and blockade of a single receptor is not sufficient to prevent the establishment of Δ^9^-THC-induced place aversion. Second, the CPP/CPA test does not directly measure the acute rewarding or aversive effects of cannabinoids. Instead, it assesses reward- or aversion-associated learning and memory captured at least 24 hours after the last Δ^9^-THC administration. As such, different receptor or neural mechanisms may underlie Δ^9^-THC-induced reduction in oICSS versus place aversion. Third, CPP/CPA experiments are infamously insensitive to subtle changes in drug reward^48, 49^. In contrast, oICSS is highly sensitive to small changes in brain reward function^32^. Last, oICSS provides a microcosm of a drug effect on a specific phenotype of neurons in a specific brain area, while place conditioning conveys the larger picture: the generally negative or positive associations an animal develops after repeated experiences to a drug. To summarize, both the oICSS and CPP assays are examining quantitatively and qualitatively distinct endpoints and a negative finding in a CPP test may not necessarily conflict with the positive finding in oICSS.

In prior work, both PPARγ and PPARα agonists are reported to decrease the reinforcing value of drugs of abuse including nicotine, ethanol, heroin, and methamphetamine^13–16^. However, the neural mechanisms underlying this action are poorly understood. Previous studies indicate that the PPARα agonists WY14643 and methOEA and the PPARγ agonist pioglitazone prevented nicotine- and heroin-induced increases in DA neuron firing in the VTA^13, 14^. In the present study, we found that dopaminergic PPAR mechanisms may directly modulate oICSS (Fig. 3C), which may explain how PPAR agonists produce therapeutic effects against drug reward.

### PPARs contribute to Δ^9^-THC-induced anxiety

Another important finding in this report is that antagonism of PPARα and PPARγ attenuated Δ^9^-THC-induced anxiety, implicating these receptors in the negative affective properties of cannabinoids. This is consistent with previous work indicating that activation of PPARα via the endocannabinoid N-palmitoylethanolamine (PEA) correlated with increases in circulating cortisol in a social stress test in humans^50^. Similarly, Domi and colleagues^44^ found that PPARγ knockout mice developed altered stress sensitivity and failed to display typical c-fos expression changes in the amygdala following stress exposure. As such, Δ^9^-THC may produce anxiety by activating PPARγ and PPARα in both GABA and glutamate neurons in the amygdala.

We note that PPARα/γ agonists or antagonists alone failed to alter basal anxiety levels, while PPARα or PPARγ antagonism only partially reduced Δ^9^-THC-induced anxiety, suggesting that in addition to PPARα/γ, other receptor (such as CB1 and CB2) mechanisms are also involved in Δ^9^-THC’s affective effects^5^. These findings mirror earlier assessments in which activation of PPARs only modulated anxiety in response to lipopolysaccharide exposure or restraint stress but did not alter basal anxiety levels^44, 51, 52^. In contrast, one report found that the same dose of the PPARγ antagonist (5 mg/kg GW9662) induced anxiety^44^. The reasons underlying these conflicting findings are unknown. More studies are required to further address this issue.

### PPARs counteract Δ^9^-THC-induced hypoactivity and catalepsy

A third important finding is that both PPARα and PPARγ modulate basal level locomotion: the agonists produced a transient increase, while the antagonists produced a robust decrease in open-field locomotion. In agreement with these findings, pretreatment with a PPARα antagonist, but not with a PPARγ antagonist, potentiated Δ^9^-THC-induced hypoactivity, suggesting that PPARα antagonism produced an additive or synergistic effect with Δ^9^-THC in open-field locomotion. In addition, pretreatment with PPARα or PPARγ antagonists also potentiated Δ^9^-THC-induced catalepsy but did not alter Δ^9^-THC-induced analgesia, hypothermia, or immobility. The former finding is consistent with a previous report indicating that pretreatment with a PPARγ agonist reduced haloperidol-induced catalepsy^53^. These findings suggest an important role of PPARα and PPARγ in modulation of locomotor behavior, but do not underlie high-dose D^9^-THC-induced tetrad effects.

In conclusion, in this study we systemically evaluated the cellular expression of PPARa and PPARg in the brain and their functional roles in the CNS effects of Δ^9^-THC. We found that PPARα and PPARγ are mainly expressed in midbrain DA neurons and in both GABA and glutamate neurons in the amygdala. Activation of PPARg inhibits DA-dependent oICSS, while blockade of PPARa and PPARg attenuates Δ^9^-THC-induced reward-attenuation (aversion) and anxiety but potentiates Δ^9^-THC-induced hypoactivity and catalepsy. These results provide novel insights regarding the role of PPARa and PPARg in cannabis action and highlight the potential utility of PPARs as new therapeutic targets for substance use disorders.

## Data Availability

The raw data in this manuscript is available upon request
